# Cultural adaptation in television technology for older adults with dementia in care settings

**DOI:** 10.3389/frdem.2022.1098446

**Published:** 2023-01-04

**Authors:** Karen Lok Yi Wong, Lillian Hung

**Affiliations:** Innovation in Dementia care and Aging Lab, School of Nursing, The University of British Columbia, Vancouver, BC, Canada

**Keywords:** cultural adaptation, television, older adults, dementia, care setting

## Abstract

**Introduction:**

There is a lack of literature on the cultural adaptation of technologies for dementia care. This paper presents an example of the cultural adaptation of a television video about drinking water for older Chinese adults with dementia in care settings, in Vancouver, Canada.

**Method:**

We refer to the cultural adaptation process (CAP) model to guide the cultural adaptation process by collecting and incorporating feedback from different parties into the culturally adapted video, including Phase 1 local consultations and information gathering, phase 2 iterative testing and adaptation, and phase 3 finalizing adaptation.

**Results:**

We also referred to the Ecological Validity Model (EVM) to present the adjustments we made to the video from the cultural adaptation. We adjusted the video on seven domains suggested by the EVM: goal, context, content, language, people, concept, and method.

**Discussion:**

We draw attention to the opportunities and challenges of the cultural adaptation of technology into a new community. Based on our lessons, we outline concrete suggestions about what aspects of, and how, cultural adaptation can be made to promote cultural inclusivity in technology development and implementation.

## 1. Introduction

Increasing use of everyday technologies among older adults with dementia supports care and quality of life (Sixsmith, 2020). One type of technology is television (TV) video programs tailored to this population. Memory Lane TV, My Life TV, and Zinnia TV are a few examples. These TV streaming videos can range from animal shows, slow-paced nature and travel videos, games, and other curated videos to stimulate multi-sensory enjoyment and support activities of daily living (ADL) care. For example, short videos can help people transition from one activity to another, from eating to hygiene/washing or grooming. The goal is to improve the health and wellbeing of people living with dementia. These videos are tailored for people with all stages of dementia to match cognitive abilities and psychosocial needs. The individual videos are typically plot-free, fun and engaging, and short, between 1 and 5 min. The messages presented are clear and easy to comprehend, considering that people with dementia may have memory loss and cognitive impairment (Breckenridge et al., 2021). Currently, from the literature, TV videos tailored for older adults with dementia are mainly used among English-speaking populations (Bjørnskov et al., 2020; Francis et al., 2020; Breckenridge et al., 2021). Practical knowledge is needed to understand how to culturally adapt such TV programs to meet the needs of other ethnic groups in the field of dementia care.

Cultural adaptation refers to the adjustment of an intervention to better suit the person's language, cultural values, and norms (Parker et al., 2022). The goal is to achieve inclusivity, ensuring that all individuals and communities, including the most disadvantaged, have access to and can use technology to enhance their quality of life (Euroageism, 2021). There are different models which guide the cultural adaptation of an intervention. The most used models are the Cultural Adaptation Process (CAP) Model and Ecological Validity Model (EVM). The CAP model is a model which suggests the steps of making the cultural adaptation of an intervention by inviting different people, from academics, and intervention providers, to intervention users, to give inputs to the cultural adaptation of the intervention and making adjustments to the intervention accordingly (Domenech Rodríguez et al., 2011). The EVM is a model to document the adjustments made to an intervention in the cultural adaptation process by referring to eight domains: language, people, metaphor, content, concepts, goals, methods, and context (Bernal et al., 1995).

There are an increasing number of interventions for older adults with dementia and their family caregivers from diverse cultural communities. For instance, the study by Parker et al. (2022) talked about turning an intervention for English-speaking family caregivers into a version for Hispanic/Latino Spanish-speaking family caregivers looking after family members with dementia in the United States. Xiao et al. (2022) adapted the English version of the World Health Organization Support for Dementia program, an educational program for family caregivers to look after people with dementia at home, into a version for Chinese family caregivers in Australia. Passos et al. (2021) turned the English version of a feeding skill checklist for caregivers of people with dementia into a version for family caregivers in Portugal. Raghuraman et al. (2017) adapted the cognitive stimulation therapy for people with dementia developed in the United Kingdom into a version for people with dementia in India. Bertrand et al. (2019) also adapted the same therapy into a version for people with dementia in Brazil.

There have been a number of cultural adaptations of technologies for use in healthcare. For example, Sit et al. (2020)'s recent publication described how their team turned the English version of a digital mental health intervention for young adults into a version for Chinese young adults speaking Cantonese in Macau and speaking Mandarin in Mainland China. In addition, Mortenson et al. (2021) discussed the cross-cultural adaptation of different technologies related to health. However, there is a lack of literature on the cultural adaptation of technology for people with dementia and their family caregivers. An example was by Efthymiou et al. (2022), which talked about the cultural adaptation of the English version of iSupport, a web-based support platform for family caregivers looking after people with dementia, into a version for family caregivers in Greece.

Canada is a multicultural country. In Canada, according to the 2016 Census of Population (Statistics Canada, 2022), 21.9% of the Canadian population is an immigrant, 17.7% of the Canadian population is a second-generation population, and 22.3% of the Canadian population is a visible minority. The percentage of the immigrant population in Canada is similar to the ones in other English-speaking developed countries, such as the United Kingdom (13%), United States (15%), New Zealand (23%), and Australia (26%) (Dow et al., 2018). British Columbia is the most culturally diverse province in Canada, while Vancouver is the most culturally diverse city in British Columbia. According to the 2016 Census of Population (Statistics Canada, 2022), 28.3% of the British Columbian population is an immigrant, 22.9% is second-generation, and 30.3% is a visible minority. On the other hand, 42.5% of Vancouver's population is an immigrant, and 48.9% of Vancouver's population is a visible minority.

This paper aims to provide a brief report on the cultural adaption process, which is a part of a larger implementation study in Vancouver, British Columbia, Canada. The larger study examines the feasibility and acceptability of implementing TV videos tailored for older adults with dementia in care settings (hospital and long-term care). This paper will use the cultural adaptation of an activity of daily living video on drinking water for Chinese people with dementia in care settings, as an example. From the authors' limited knowledge, this paper was the first to discuss a video's cultural adaptation for people with dementia.

## 2. Method

Our research team consisted of two patient partners (i.e., people with lived experience of dementia) and two family partners (i.e., people with lived experience looking after people with dementia), three clinical partners (site champions) (i.e., one rehabilitative staff at the hospital site, one nurse leader and one recreation therapist at the long-term care site), one principal investigator (PI, a university professor in Nursing) and three research trainees (undergraduate and graduate students in nursing and social work), and four industry partners (i.e., developers of the TV video company). Our team members have diverse cultural backgrounds, including Caucasian, Chinese, Filipino, and German.

The CAP model guides our processes on the cultural adaptation process for Chinese older adults with dementia in care settings. We also utilize the EVM model to inform the domains of the content modifications to ensure language, persons, content, concepts, goals, methods, and contexts in the video program are acceptable, relevant, comprehensible, and complete for Chinese older adults living with dementia.

### 2.1. Settings and participants

The study sites involve a long-term care home and a geriatric hospital unit in Vancouver. In both sites, the average age of residents/patients was 80 years old and above. About 80% of residents/patients had dementia. Both study sites have a high proportion of residents/patients from the Chinese community. The long-term care home is culturally specific for people of the Chinese community. The settings were selected for the study because we had the leaders' support, and the teams were willing to collaborate with us in implementing the TV program. A convenient sampling method was used to recruit staff in all disciplines to participate in focus groups. The clinical partners (site champions) in the research team helped to promote and recruit staff participants.

### 2.2. Procedures

There were three phases of cultural adaptation from February 2022 to November 2022: (1) Local consultations and information gathering, (2) Iterative testing and adaptation, and (3) Finalizing adaptation (see Table 1).

**Table 1:** Phases of cultural adaptation of the video guided by the cultural adaptation process (CAP) model.

**Phase**	**Description**
1	Local consultations and information gathering
2	Iterative testing and adaptation
3	Finalizing adaptation

#### 2.2.1. Phase one: Local consultations and information gathering

We conducted five focus groups. There were 23 staff participants from four disciplines (see Table 2). There were three to eight staff participants in each focus group. Each focus group lasted for about 30 min. All staff were bilingual in English and Chinese. All except one focus group were conducted in English. The focus group not conducted in English was in Chinese because staff participants in this group suggested that they could better express their views in Chinese than in English. The focus groups were conducted in person or by zoom by two bilingual research trainees trained to facilitate focus groups. The focus groups were audio-recorded and transcribed. A research trainee also took notes. Staff participants were asked about their experience meeting psychosocial needs with patients and residents, followed by their perspectives about using TV videos in their settings. Feedback was sought about resources and adaptations needed. The domains of the EVM informed the discussion process. For example, we focused on the type of videos, delivery methods, culturally appropriate content, etc.

**Table 2:** Background information of staff participants (*n* = 23).

**Category**	**Sub-category**	**Number**
Site	Long-term care home	12
	Geriatric acute care unit	11
Discipline	Nurses and care aide	12
	Recreation therapist and recreation aide	7
	Rehabilitation	3
	Administration	1

#### 2.2.2. Phase two: Iterative testing and adaptation

A family partner with a Chinese background was invited to film a drinking water video based on the feedback in Phase One. In our regular research meetings *via* zoom, the research team reviewed and discussed the iterations of the video produced. In addition, a Chinese subtitle was added to the video. These research meetings were an hour and recorded. Notes were taken by one of the research trainees during the meetings. Different team members gave their input about the cultural adaptation of the videos. The meetings were facilitated by one of the research trainees. EVM discussed essential domains: goal, context, content, language, people, concept, and method. See details of staff inputs presented in the results section. New videos were produced based on the staff input and team discussion to meet the users' needs. An example of a new video can be found: (at https://www.zinniatv.com/videos/drink-water-in-cantonese).

#### 2.2.3. Phase three: Finalizing the culturally adapted video

The research team tested the new video with residents/patients in the two study sites. We are still in the process of testing. Up to now, we have shown the video to five participants. They were all Chinese women who spoke Cantonese with moderate to severe dementia. We observed, tape-recorded and took field notes of their comments and facial expressions. We conducted a preliminary analysis of the observation and field notes. Firstly, based on the participants' comments, we learned that the older Chinese adult in the video stood out to many participants. They either commented on her or gave positive facial expressions when they saw her. Second, one participant commented on the people drinking water in the video, “They were drinking so quickly.” As a clinical partner remarked, “older adults with dementia are encouraged by staff in care settings to drink slowly to avoid choking risk.” Third, there was a scene in the video in which a person drank water directly from a tap. When a participant saw this scene, she said, “I would not do that.” Research team members who self-identified as coming from the Chinese community explained that Chinese people, especially older adults, seldom drink water directly from the tap and usually boil the water before drinking. The participant made a comment because the scene lacked cultural relevancy. We are now involving the industrial partners in the feedback discussion and continue to use the lessons learned in the adaption process to refine and develop new videos.

### 2.3. Data analysis

Our team conducted a reflexive thematic analysis based on the inputs from the research team documented in meetings notes, input from the staff of study sites in focus group notes and transcripts, and fieldnotes from observation of residents/patients using the Chinese version of the video (Braun et al., 2019). A research trainee did the initial analysis. She used NVivo to assist with the process. She first read the notes and transcripts. After that, she coded the data. Next, she grouped codes into categories and categories into themes. She made constant comparisons of data, codes, categories, and themes. PI checked the codes. Both the research trainee and PI read the transcripts and agreed on the codes, categories and themes. When they had disagreements about the codes, categories, and themes, they discussed them until they reached a consensus. Finally, the research trainee presented the analysis to the research team members. The goal was to avoid bias by getting feedback from people from diverse backgrounds and disciplines to ensure rigor and by getting comments from patient and family partners who have lived experience as well as clinical partners who have frontline experience working with people with dementia. Patient and family partners challenged some “norms.” For instance, the original video had a Chinese person and an older adult. However, our patient partner raised that our target population might not be able to relate to the video because there was no older Chinese adult. This patient partner challenged people's assumptions of thinking of identities separately and raised the importance of considering the intersection of identities. In our case, this referred to the intersection of race and age. Team members gave feedback. Industry partners further revised the video accordingly.

Both deductive and inductive approaches guided the analysis: We did data analysis deductively and were sensitized by the concepts in the literature. In our case, concepts in the literature referred to the domains of EVM. For example, when we read the data on a patient partner talking about how older adults whose English was not their first language might have challenges understanding the original English version of the video, we went back to the “language” domain of EVM and compared and discussed the concepts to code the segment. We also did data analysis inductively, always remained open and let new concepts emerge from the data. For instance, when we read the data on a care aide talking about how older Chinese adults prefer warm or hot water because of the traditional belief that cold water is not good for health, we coded “cultural belief” for this data.

### 2.4. Ethical consideration

The UBC Ethics Board approved the study: H22-00739. Written consent forms were obtained from participants.

## 3. Results

This section will present the cultural adaptation we made to the video with reference to the EVM (see Table 3). The following will present how the cultural adaptation responded to each domain.

**Table 3:** Cultural adaptation of the video according to EVM domains.

**Domains of ecological validity model (EVM)**	**Cultural adaptation of the video**
Goal	Set the goal of the cultural adaptation to “turn the original video into a video which matched the language and beliefs of the Chinese older adults with dementia in care settings.”
Context	Remember that the video will be used in care settings with Chinese older adults with moderate to severe dementia
Content	Add a few scenes in the video where a person giving simple instructions on drinking water
Language	Change the title of the video to Chinese
	Decide the instruction on the drinking water will be in Chinese
People	Have an older Chinese adult appear in the video to give the instruction
Concept	Add a few scenes in the video that a person was drinking water using a cup, which implies drinking warm or hot but not cold water, considering the traditional Chinese belief that drinking cold water is not good for health
Method	Deliver the video *via* both smart TV and tablet, considering the video will be used in care settings

### 3.1. Goal

The goal refers to what we hope to achieve through the cultural adaptation of the video. According to our clinical partners (site champions), many older adults with moderate to severe dementia in care settings, including those from the Chinese community, have challenges drinking water alone due to cognitive impairment. It would be helpful if the self-care videos could encourage older adults to drink water. The feedback also indicated that Chinese older adults with dementia might not understand the encouragement if the encouragement does not match the Chinese older adults' language and beliefs. Therefore, it would be much more effective if adaptation could be done to the video to match the language and beliefs of older Chinese adults with dementia in care settings to encourage them to drink water.

### 3.2. Context

Context means the conditions under which the culturally adapted video will be used. As suggested, the video will be used in care settings with Chinese older adults with moderate to severe dementia. The context guided our cultural adaptation, which will be explained in other domains of the EVM below. Our research team members referred to the context when discussing the video's cultural adaptation.

### 3.3. Content

Content refers to what will be included in the culturally adapted video. A care aide staff of the long-term care study site shared in a focus group that, according to her practice experience, when she was encouraging an older adult with dementia to drink water, she found it helpful to stand in front of the older adult and give simple instructions on drinking water constantly until the older adult finishes the water. She hoped the culturally adapted video could have something similar, having a person in front of the screen and giving simple instructions on drinking water. The original video encourages older adult audiences with dementia to drink water by showing different people drinking water. Soft background music but no voice-over or subtitles give instructions on drinking water. Based on the sharing of this care aide, our team decided to include a person providing simple instructions on drinking water in the video. This led to a discussion in the research team about the language to use to give the instructions and the person giving the instructions, which will be explained below.

### 3.4. Language

Language means the language that will be used in the culturally adapted video. A patient partner in his 70s who self-identified as a Filipino Canadian raised in a team meeting that it was essential to pay attention that the older adult audience with dementia whose English was not their first language might not know English or may have lost their English ability due to dementia. Thus, they might not understand if the instruction was in English. A family partner in her 80s who self-identified as coming from the Chinese community and had family, work, and volunteer experience with Chinese older adults with dementia added that the language barrier was a challenge to many Chinese older adults from her observation when she was caring for them. The title “drink water” in the original video was in English. We decided to change it to Chinese. Also, as suggested, our team added a person giving simple instructions on drinking water in the video. The instructions were in Chinese based on feedback from the patient and family partners. An industry partner further asked in the team meeting whether the instructions should be in Cantonese or Mandarin, the two most widely spoken Chinese languages in Vancouver (City of Vancouver, 2022). A site partner of the long-term care home responded that there are more residents who talk in Cantonese than in Mandarin in their care home. She further explained that although the proportion of people in Vancouver who speaks Mandarin is increasing, among the older Chinese population, especially the oldest old, there are still more Cantonese than Mandarin speakers. These older Chinese adults came to British Columbia after the Second World War from the Canton province of mainland China or in the 1990s from Hong Kong. The site partner of the geriatric hospital unit also added that many older Chinese patients speak Cantonese. Based on the inputs of the clinical partners (site champions), our team decided to add a person giving instructions in Cantonese in the video.

### 3.5. People

**Figure 1:**
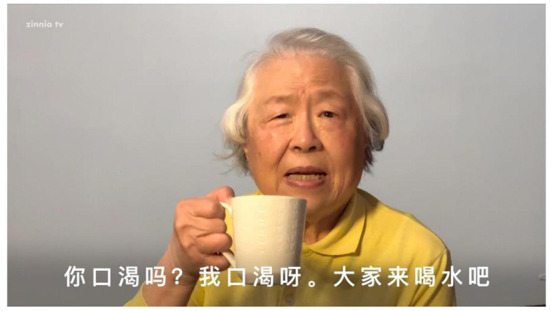
The scene in the Chinese version of the drinking water video.

People refer to the people who appeared in the video. In the original video, there were people from diverse cultural backgrounds and a wide age range. However, there was no older Chinese adult. Our patient partner raised in a team meeting that older adults with dementia may not relate to the videos if they cannot find people who look like them. A recreation aide staff of the long-term care home study site also made this point in a focus group. Based on this feedback and following our previous discussions on having a person give simple drinking water instructions, our team planned to have an older Chinese adult appear in the video to provide the education (please see Figure 1).

### 3.6. Concept

Concept refers to traditional beliefs. The original video showed people drinking water using a glass or a plastic bottle. The reason for using the glasses and plastic bottles is that older audiences with dementia could see the water in the containers, and it was hoped that seeing the water could encourage the audience to drink water. However, a care aide staff of the long-term care home study site raised in a focus group that using a glass and plastic bottle implied that the people in the video were drinking cold water. From her experience working with older Chinese adults, many preferred warm or hot water because of the traditional belief that cold water is not good for health. She suggested that the video should indicate that people are drinking warm or hot but not cold water. A research trainee who is a nurse made a similar comment in a research team meeting according to her work experience with older Chinese adults. Based on these comments, our research team added a few scenes in the video where a person was drinking water using a cup, which implies drinking warm or hot but not cold water.

### 3.7. Method

Method means the method to deliver the video. Our research team initially only thought about using the video *via* a smart TV. The clinical partners (site champions) of both study sites raised in a team meeting that not all older adults in care settings have a smart TV in their rooms. Even though both study sites have smart TVs in their common areas, the clinical partners (site champions) explained that sometimes it could be challenging for staff to bring older adults to the common areas. Also, some older adults in care settings have a visual impairment and might not be able to see what is shown on the TV unless they are very close to it. Therefore we considered possible options to display videos for adaptability. For example, tablets are more convenient to bring to the bedside. As requested by staff, mobile stands were added to the iPad/ tablets so they could wheel the videos close to the patient or resident. A mobile app was developed for quick access and easy video selection.

## 4. Discussion

In this paper, we demonstrated an example of a cultural adaptation of a television video about drinking water for older Chinese adults with dementia in care settings. We used the CAP model to guide the process, and the phases included Phase 1: local consultations and information gathering, phase 2: iterative testing and adaptation, and phase 3: finalizing adaptation. We also used the EVM to show our cultural adaptation to the video. By referring to EVM, our themes of cultural adaptations fell into the seven domains of EVM: goal, context, content, language, people, concept, and method. We compared our results with previous literature on cultural adaptation of healthcare technologies or older adult interventions. Not all previous literature used models to guide the presentation of cultural adaptation made (Xiao et al., 2022; Raghuraman et al., 2017; Bertrand et al., 2019; Passos et al., 2021). Theoretical models can provide useful guidance on data analysis and presentations of results (Sit et al., 2020; Parker et al., 2022). The CAP and EVM models helped us to think comprehensively and consider relevant constructs in evidence-based domains, which enhanced the rigor of our cultural adaptation work.

### 4.1. Strengths of the cultural adaptation

This paper has several strengths. First, this is the first paper to depict the cultural adaptation process on TV videos for dementia care. Our case example offers encouragement for more cultural adaptation studies in this area and supports older adults with dementia from diverse cultural communities. A culturally adapted program can increase relevance and decrease the risk of technology abandonment. It is also an ethical obligation to respect diverse cultural values and identities. Second, using the CAP model and EVM, this paper shows how to make cultural adaptations to technologies in dementia care step by step. The collaborative work with clinical, patient and family partners, along with the industry, build trust. Finally, we involved patient partners, who are people with lived experience with dementia, in our research team. There are limited cultural adaptation studies on dementia care which involve people with dementia in the research team.

One lesson our team learned is that to make a cultural adaptation of technology for older adults with dementia, it is important to have a team consisting of members coming from diverse backgrounds, bring them together, and create an environment for them to discuss. So that we can have input from different perspectives. This echoed the previous literature on cultural adaptation studies (Sit et al., 2020). In our case, clinical partners (site champions) and staff contributed their knowledge about working with older Chinese adults with dementia. In addition, family and patient partners contributed their expertise from their lived experiences living with dementia or providing care.

Another lesson we learned is that when we make a cultural adaptation, we need to consider not only the culture and language of the users of the intervention but also other elements of the users, such as their age, abilities, and surrounding environment, and how these elements intersect and influence the cultural adaptation. For example, in our case, the video users were not only Chinese but also older adults with dementia in care settings.

### 4.2. Limitation of the cultural adaptation

Our cultural adaptation has several limitations. First, we have not evaluated the effectiveness based on residents' responses. We are still making a few more culturally adapted videos for diverse groups of older adults with dementia in care settings. Therefore, we have more work to do in terms of data collection and analysis. However, we already had feedback from patient partners who have lived experience with dementia and family partners who have experience looking after family members with dementia, as well as clinical partners (site champions) and staff with work experience with older Chinese adults with dementia. This brief report offers preliminary findings. In the next paper, we will report the effectiveness of our culturally adapted video. Second, our staff participants were from two large Vancouver facilities serving a predominance of Chinese patients or residents. The acceptability of TV videos remains unknown for other aged care settings with a different demographic mix. However, these Chinese-tailored videos may be transferable to similar aged-care settings in Toronto or other comparable cities. Third, we did not include dementia and language experts in the adaptation process to provide additional feedback on the modifications. Future studies should consist of expert perspectives, including people with expertise in psychology and communication, which may complement lived experience expertise for robust outcomes.

## 5. Conclusion

People with dementia are heterogenous with diverse cultural backgrounds. This study provides a case example to demonstrate how to involve patient partners and relevant stakeholders in an active partnership for adapting TV video development and implementation in aged care settings to achieve inclusivity. Future research in implementation science should further investigate how to carry out cultural adaptation to meet the diverse needs of people with dementia to promote inclusivity.

## Data availability statement

The raw data supporting the conclusions of this article will be made available by the authors, without undue reservation.

## Ethics statement

The studies involving human participants were reviewed and approved by UBC Ethics Board. The patients/participants provided their written informed consent to participate in this study. Written informed consent was obtained from the individual(s) for the publication of any potentially identifiable images or data included in this article.

## Author contributions

KW conducted investigation, managed the data, conducted data analysis, drafted the manuscript, and handled project administration. LH conceptualized the project, conducted investigation, conducted data analysis, reviewed and edited the draft, and supervised the study. Both authors contributed to the article and approved the submitted version.
